# Efficiency of Combining Strains Ag87 (*Bacillus megaterium*) and Ag94 (*Lysinibacillus* sp.) as Phosphate Solubilizers and Growth Promoters in Maize

**DOI:** 10.3390/microorganisms10071401

**Published:** 2022-07-12

**Authors:** Luana Rainieri Massucato, Suelen Regina de Araújo Almeida, Mayara Barbosa Silva, Mirela Mosela, Douglas Mariani Zeffa, Alison Fernando Nogueira, Renato Barros de Lima Filho, Silas Mian, Allan Yukio Higashi, Gustavo Manoel Teixeira, Gabriel Danilo Shimizu, Renata Mussoi Giacomin, Ricardo Cancio Fendrich, Marcos Ventura Faria, Carlos Alberto Scapim, Leandro Simões Azeredo Gonçalves

**Affiliations:** 1 Agronomy Department, Universidade Estadual de Londrina (UEL), Londrina 86051-990, PR, Brazil; lrmassucato@gmail.com (L.R.M.); suelen.araujo.16@gmail.com (S.R.d.A.A.); alllisonfernando@gmail.com (A.F.N.); silasmian@hotmail.com (S.M.); allanhigashi@gmail.com (A.Y.H.); shimizu@uel.br (G.D.S.); 2 NODUSOJA, Colombo 83407-330, PR, Brazil; mayara@nodusoja.com.br (M.B.S.); ricardo@nodusoja.com.br (R.C.F.); 3 Microbiology Department, Universidade Estadual de Londrina (UEL), Londrina 86051-990, PR, Brazil; moselamirela@gmail.com (M.M.); gustavomanut@gmail.com (G.M.T.); 4 Agronomy Department, Universidade Estadual de Maringá (UEM), Maringá 87020-900, PR, Brazil; douglas.mz@hotmail.com (D.M.Z.); delimafilho.renato@yahoo.com (R.B.d.L.F.); cascapim@uem.br (C.A.S.); 5 Biology Department, Universidade Estadual do Centro Oeste (Unicentro), Guarapuava 85015-430, PR, Brazil; giacomin.rm@gmail.com; 6 Agronomy Department, Universidade Estadual do Centro Oeste (Unicentro), Guarapuava 85015-430, PR, Brazil; ventura_faria@yahoo.com.br

**Keywords:** *Zea mays* L., microbial inoculants, phosphorus solubilization, phosphorus use efficiency

## Abstract

Increasing phosphorus (P) use efficiency in agricultural systems is urgent and essential to significantly reduce the global demand for this nutrient. Applying phosphate-solubilizing and plant growth-promoting bacteria in the rhizosphere represents a strategy worthy of attention. In this context, the present work aimed to select and validate bacterial strains capable of solubilizing phosphorous and promoting maize growth, aiming to develop a microbial inoculant to be used in Brazilian agriculture. Bacterial strains from the maize rhizosphere were evaluated based on their ability to solubilize phosphate and produce indole acetic acid. Based on these characteristics, 24 strains were selected to be further evaluated under laboratory, greenhouse, and field conditions. Among the selected strains, four (I04, I12, I13, and I17) showed a high potential to increase maize root growth and shoot P content. Strains I13 (Ag87) and I17 (Ag94) were identified by genomic sequencing as *Bacillus megaterium* and *Lysinibacillus* sp., respectively. These strains presented superior yield increments relative to the control treatment with 30% P. In addition, combining Ag87 and Ag94 resulted in even higher yield gains, indicating a synergistic effect that could be harnessed in a commercial inoculant for Brazilian agriculture.

## 1. Introduction

Phosphorus (P) is an integral component of several important compounds in plant cells and is essential for plant growth and development [1]. However, a large proportion of the P present in soils is not available to be absorbed by plants. In calcareous soils, Ca^2+^ increases P precipitation, while in acidic soils, Al^-^ and Fe^-^ oxides are responsible for P immobilization [2]. In addition, the low mobility of this nutrient in the soil solution hinders its acquisition mechanisms, as they depend on direct interception by roots.

An alternative to minimize P deficiency in acidic soils is to apply correctives and phosphate fertilizers, adapting the soil to the plant. Phosphate fertilizer, derived mainly from phosphate rocks, is widely used in agriculture and has contributed significantly to food production, a pillar of food security [3]. However, only 15% of the P applied is absorbed by plants, and the addition of this inorganic fertilizer in excess can cause environmental problems, leading to the accumulation of heavy metals in soil, contamination of groundwater, and eutrophication of water sources [4]. Furthermore, a significant proportion of phosphate rock reserves for mineral extraction are concentrated in a relatively small area, mainly in Morocco and Western Sahara, making many countries dependent on imports to supply their P demands [5]. For instance, in Brazil, approximately 4.5 million tons of inorganic fertilizers are imported per year [6].

Increasing P utilization efficiency in agricultural systems is fundamental to substantially reducing its demand. An important strategy is using microbial inoculants with P solubilization activity in the soil [7,8,9]. These microorganisms use several solubilization and mineralization mechanisms that convert inorganic and organic P, respectively, into a bioavailable form, facilitating absorption by plant roots. Moreover, some of these microorganisms also demonstrate potential as plant growth promoters and biocontrol agents against plant pathogens [2,7].

Many microorganisms, including bacteria, fungi, actinomycetes, and algae, exhibit the ability to solubilize and mineralize P. Among bacteria, strains of the genera *Pseudomonas*, *Mycobacterium*, *Micrococcus*, *Bacillus*, *Flavobacterium*, *Rhizobium*, *Mesorhizobium*, and *Sinorhizobium* have been reported to solubilize P [7,10,11]. These bacteria can make P available to plants through several mechanisms, some more related to enzymatic processes (phytases and/or phosphatases), while others involve cellular physiology, with the extrusion of H^+^ ions and release of organic acids from microbial metabolisms [12].

Phosphate-solubilizing bacteria (PSB) have great potential in regions with phosphorus deficiency, such as the Brazilian Cerrado. Under natural conditions, Cerrado soil is characterized by low pH and low nutrient fertility, especially P. In addition, the soil of these regions has a high capacity for P fixation, mainly due to the high contents of iron and aluminon oxide. Despite these natural restrictions, this region was responsible for 78% of Brazilian maize production in the 2020/2021 harvest [13]. In this context, this study aimed to select and validate bacterial strains that solubilize phosphorus and promote maize growth, aiming to develop a microbial inoculant for Brazilian agriculture.

## 2. Materials and Methods

### 2.1. Screening of Bacterial Isolates—Phosphate Solubilization and Indole Acetic Acid Production

Ninety-two bacterial strains from the maize rhizosphere were evaluated for their ability to solubilize phosphate and produce indole acetic acid (IAA). These strains were isolated from maize rhizospheric soil collected in the municipality of Italva, Rio de Janeiro, Brazil. For P solubilization, 100 µL of each strain was plated in the National Botanical Research Institute’s phosphate growth medium (NBRIP) [14], containing inorganic iron phosphate (FePO_4_.2H_2_O). The inoculated plates were incubated at 25 °C for 10 days, and microorganism growth was monitored daily. The isolates were selected by growth and/or formation of a halo, indicating the solubilization of the compound in the culture medium. In the control treatment, the plates were inoculated with sterile saline solution (0.85%).

IAA production was determined according to the Salkowski colorimetric assay, as described by Sawar et al. [15]. The cell-free culture supernatants obtained in DYGS liquid medium supplemented with tryptophan (100 µg mL^−1^) were incubated for 48 h at 28 °C and 180 rpm in an orbital shaker (Tecnal—TE 422, Piracicaba, São Paulo, Brazil). The concentration of IAA produced by each strain was estimated by measuring its optical density at 530 nm and using an IAA standard curve. Based on the results of P solubilization and IAA production, 26 bacterial strains were selected for growth promotion assays in maize under greenhouse conditions.

### 2.2. Preparation of Bacterial Isolates

Strains stored in cryovials containing liquid Tryptic Soy Broth (TSB) and glycerol in a 2:1 ratio at −80 °C were activated in Petri dishes containing LBA (Luria Bertani Agar, Neogen Corporation, United States) culture medium at 28 °C for 24 h. A pre-inoculum of each strain was prepared from pure colonies suspended in saline solution (0.85% sodium chloride), with turbidity adjusted to 0.5 in the McFarland nephelometric standard (1.5 × 10^8^ CFU/mL). Thirty uL of these bacterial suspensions were transferred to 125 mL Erlenmeyer flasks containing 30 mL of Ag/02 culture medium (g L^−1^: glucose 15.0, sucrose 10.0, yeast extract 10.0, micronized soy protein 10.0, KH_2_PO_4_ 1.5, MgSO_4_ 0.5, MnSO_4_ 0.5, CaCl_2_ 1.5, and pH 8.0) and incubated at 30 °C for 18–20 h at 200 rpm in an orbital shaker (Tecnal—TE 422, Piracicaba, São Paulo, Brazil) for inoculum production. For fermentation, 1000 mL Erlenmeyer flasks containing 400 mL of Ag/02 were inoculated with a 4 mL aliquot of the inoculum and incubated at 30 °C for 72 h at 200 rpm. After fermentation, the production concentration was adjusted to 2.0 × 10^9^ CFU/mL.

### 2.3. Laboratory and Greenhouse Tests

Maize seeds of the cultivar P30F53 (Pioneer^®^, São Paulo, Brazil) were inoculated with bacterial strains at a 2 × 10^9^ CFU/mL concentration, constituting 26 treatments. The following treatments were used as controls: no inoculation, Biomaphos (*Bacillus subtilis* strain CNPMS B2084 and *Bacillus megaterium* strain CNPMS B119), and Nodugram (*Azospirillum brasilense* strain Abv5). The seeds were treated at a dose of 100 mL/60,000 seeds.

After inoculation, 15 seeds of each treatment were placed on germination paper with sterilized distilled water in a growth chamber (25 ± 2 °C and 70% relative humidity). For this experiment, four repetitions were used. Ten days after sowing, five seedlings were selected for the evaluation of average root diameter (ARD), root surface area (RSA), and root length (RL) by scanning them in a scanner with a resolution of 300 dpi. First the images were treated and analyzed by GiaRoots software [16]. Then, the shoot and root systems of the seedlings were kept in an air forced oven at 60 °C for 72 h. Then, shoot and root dry mass (SDM and RDM, respectively, in g) were determined. The experiment was conducted in a completely randomized design.

For the greenhouse tests, two experiments were performed, one using sand as the substrate and the other using sand and soil (3:1). Twenty-eight treatments were evaluated, comprising 26 bacterial strains and 4 controls (Biomaphos, Nodugram, insoluble P, and no P). Seeds of the hybrid P30F53 (Pioneer^®^) were inoculated and sown in 970 mL pots containing sterilized sand or sand with soil collected in a cultivation area at School Farm, Universidade Estadual de Londrina (UEL). Two seeds were sown per pot, and, ten days after sowing (DAS), thinning was performed, keeping only one plant per pot. The experiments were carried out in a completely randomized design with six repetitions.

The treatments were irrigated with Hoagland’s nutrient solution [17], modified by Magnavaca et al. [18]. A volume of 100 mL of the solution was applied every three days. The plants were removed 28 DAS and subjected to evaluation of the stem diameter. Subsequently, shoots and roots were separated and air-dried in an oven with forced ventilation for 72 h to measure SDM and RDM. After drying, shoots were ground and evaluated for P content. To determine the P content in the grains and shoots, the samples were dried in an oven at 70 °C for 72 h and ground with Willey MA340-type knives (Piracicaba, São Paulo, Brazil). Then, 0.1 g aliquots were digested in nitroperchloric solution (HNO_3_:HClO_4_) according to Malavolta et al. [19]. The P content was determined by the molybdenum blue spectrophotometric method [20], and the readings were performed in an Agilent 8453 spectrophotometer (Agilent Technologies, Santa Clara, CA, USA) at a wavelength of 660 nm.

### 2.4. Genomic Sequencing of the Strains Ag87 and Ag94

Based on the results obtained in the laboratory and greenhouse, two strains were selected for field trials, which were cataloged in the AgBio microorganism bank and named Ag87 and Ag94. For complete genome sequencing of these strains and species identification, they were cultured in LB medium at 150 rpm at 28 °C for 48 h. DNA extraction was performed using a PureLinkTM Microbiome DNA Purification kit (Invitrogen, Thermo-Fisher Scientific, Waltham, MA, USA). DNA integrity was verified using a 1% agarose gel, and the DNA was quantified by spectrophotometry in a NanoDrop 2000/2000 c (ThermoFisher Scientific, Wilmington, DE, USA). The sequencing was performed on the Illumina MiSeq platform of the company SuperBAC, Mandaguari, Paraná, Brazil.

The quality of the readings and the cut-off parameters were considered and chosen using FastQC [21]. Using the Trimmomatic program [22], the raw readings were filtered based on the parameters defined by FastQC. After the filters, the quality of the readings was analyzed again to check if the chosen parameters were adequate. A series of de novo assemblies were performed in two software programs (SPA-des and IDBA hybrid) [23], testing different assembly parameters and comparing them with each other in the QUAST program [24]. According to the reference genome provided in QUAST, key metrics such as total alignment size, number of contigs, largest contig, N50 values, and gene numbers were used to choose the best assembly. Using the CONTGuator web server, best-assembled contigs were aligned with the genomes of *Bacillus megaterium* DSM319 and *Lysinibacillus agricola* FJAT-51161 for strains Ag87 and Ag94, respectively, to generate the scaffolds. Gaps were filled manually, mapping reads using Bowtie2 and filling gaps using CLC Genomics Workbench 12 GUI [25]. The genome start point was determined by comparison with a reference strain genome, assuming the dnaA gene as the first gene.

The genomes of strains Ag87 and Ag94 were represented circularly and compared with other reference genomes using BRIG software (BLAST Ring Image Generator) [26]. Genomic comparisons were performed with the program Gegenees [27]. SplitsTree [28] was used to represent phylogenetic relationships in a tree. For species determination, ANI (average nucleotide identity) and dDDH (digital DNA–DNA hybridization) were performed among other *Bacillus* spp. and *Lysinibacillus* sp. using OrthoANI [29].

### 2.5. Field Trials—Harvest (2020/2021)

For the tests under field conditions, seeds of the maize cultivar P3340VYHR (Corteva^®^, Indianapolis, IN, USA) were used. The seeds were treated with biological products (Ag87, Ag94, Ag87 + Ag94, and the commercial product Biomaphos) in plastic bags using a dose of 100 mL/60,000 seeds.

The experiments were carried out during the 2020/2021 summer (first) harvest in the municipalities of Londrina, Maringá, Guarapuava, and Entre Rios do Oeste, state of Paraná, Brazil. The physical-chemical analyses of the soils and other characteristics related to the evaluation sites are presented in Table S1.

The experimental design adopted was complete randomized blocks with four repetitions. The plots consisted of eight rows of 6 m in length with a spacing of 0.45 m between rows and five plants per meter. Before setting up the experiment, the areas were fertilized with 25 kg P_2_O_5_ ha^–1^, 60 kg K_2_O ha^–1^, and 21 kg N ha^–1^. The amount of P_2_O_5_ applied in the experiments was 30% of the standard phosphate fertilization recommended for maize. The topdressing fertilization in the crop was carried out with 120 kg N ha^–1^ applied at the V6 development stage.

Three representative plants from each plot of the experiments were collected at the physiological maturation stage. The determination of P content in the grains and shoots was carried out as described in Section 2.3. Grain yield (GY, in kg ha^–1^ and 13% moisture) was obtained after manual harvesting and mechanical threshing of the plants in the six central rows of each plot. The components of P use efficiency (PUE) were determined according to Moll et al. [30]. P uptake efficiency (PUpE, in g of absorbed P per g of applied P) was calculated by the ratio between total plant P and P available to the plant. P utilization efficiency (PUtE, in g of grains produced per g of total plant P) was determined by the ratio between grain dry biomass and the amount of total plant P, while PUE (in g of grains produced per g of applied P) was calculated by the product of PUpE and PUtE.

### 2.6. Field Trials—Second Harvest (2021)

The experiments were carried out during the second harvest in the municipalities of Londrina and Maringá, state of Paraná, and in the municipalities of Itiquira, Sorriso, and Sapezal, state of Mato Grosso. The physical-chemical analyses of the soils and other characteristics related to the evaluation sites are presented in Table S2. The experimental design adopted was complete randomized blocks with four repetitions. The plots consisted of eight 6 m long rows with 0.45 m spacing between rows and five plants per meter for the Londrina and Maringá experiments, while for the Mato Grosso experiments, three plants per meter were used. In Londrina and Maringá, the areas were fertilized at sowing with 25 kg P_2_O_5_ ha^–1^, 60 kg K_2_O ha^–1^, and 21 kg N ha^–1^. Then, topdressing fertilization of 120 kg N^–1^ ha was applied at the V6 development stage. In Mato Grosso, the areas were fertilized with 51 kg K_2_O ha^–1^ at the V1 stage and 100 kg N ha^–1^ at V4. Concerning phosphorus, 13 kg P_2_O_5_ ha^–1^ was applied before sowing in the 30% P control and biological treatments. For the 50 and 100% P controls, 22 and 45 kg P_2_O_5_ ha^–1^ was applied, respectively. Grain yield (GY, in kg ha^–1^ and 13% moisture) was obtained after manual harvesting and mechanical threshing of the plants in the six central rows of each plot.

### 2.7. Data Analysis

The agronomic data were subjected to analysis of variance, Scott–Knott mean cluster analysis (laboratory and greenhouse assays), and Tukey’s test (field assays). In addition, the greenhouse data were subjected to correlation and multivariate analysis using principal component analysis (PCA) and UPGMA hierarchical clustering based on standardized Euclidean distance. These analyses were performed by the R program using the packages “AgroR” [31], “factoMiner” [32], “pheatmap” [33], and “ggplot2” [34].

## 3. Results

### 3.1. Screening of Bacterial Strains

Using NBRIP medium for cultivation, 42 strains were capable of solubilizing phosphate and producing IAA with values ranging from 0.15 to 18.30 µg mL^−1^. Based on the traits of phosphate solubilization and IAA production, 24 strains were selected for laboratory and greenhouse experiments.

In the germination paper experiment, a significant effect of treatments was observed for all of the analyzed traits. The coefficient of variation ranged from 8.12 (RL) to 14.15 (SDM). For ARD, 17 bacterial strains obtained higher values than the control treatment, ranging from 0.0812 to 0.1054 mm (Table S3). For RSA, 13 strains obtained better results than the control, with emphasis on strains I04 and I10, while for RL, 11 strains were superior to the control, especially strains I04, I10, and I11. For DRM, the strains that stood out were I10, I13, and I25, with 65% more dry root mass, on average, than the control. Moreover, a total of 18 bacterial strains obtained higher values for this trait than the control. For SDM, the strains that stood out were I09, I20, and I26, with an average increase of 19% compared to the control.

For the greenhouse experiment, a significant effect was observed for most traits, except for SDM (experiment sand:soil, Exp_SS). The coefficient of variation ranged from 6.15 (RDM—experiment soil, Exp_S) to 12.15 (SDM—Exp_SS). For the experiment in the sand, strains I04, I05, I13, and I17 obtained the highest values for RDM, with increments of 58, 51, 67, and 53%, respectively, in relation to the control (Pinsol) (Table S4). These strains also obtained higher values of RDM in the sand:soil experiment, with increments of 33, 25, 30, and 32%, respectively, in relation to the control (Pinsol). For this experiment, nine strains obtained values higher than the control. For SDM, no difference was observed between the treatments and the control (Pinsol). For shoot P content, no differences were observed for treatments compared to the control (Pinsol) in the sand experiment. By contrast, in the sand:soil experiment, the Biomaphos, I04, I10, I13, I17, and I22 treatments led to 26, 15, 21, 16, 25, and 11% more shoot P content than Pinsol.

**Figure 1:**
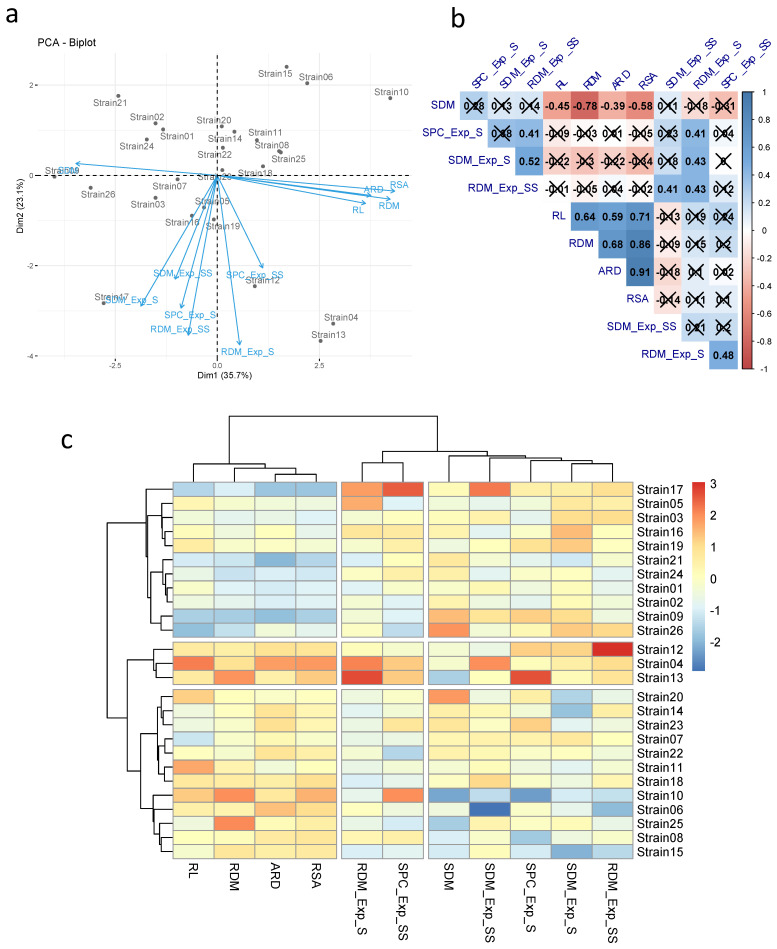
Multivariate analysis and correlation among traits evaluated in maize experiments (germination paper and greenhouse) with seeds inoculated with different phosphorus-solubilizing bacteria. (**a**) Principal component analysis; (**b**) Pearson correlation; and (**c**) UPGMA hierarchical grouping. (×: No significant effect). (ARD: average root diameter, RSA: root surface area, RL: root length, RDM: root dry mass, SDM: shoot dry mass, and SPC: shoot phosphorus content. Exp_S: greenhouse experiment—sand substrate, Exp_SS: greenhouse experiment—sand:soil substrate (3:1)).

According to the principal component analysis, the first two components explained 58.8% of the total variation (PCA1 and PCA 2 with 35.7 and 23.1%, respectively) (Figure 1a). The traits related to the root system (RL, RDM, ARD, and RSA) were correlated with each other (Figure 1a,b). However, they showed a negative correlation with SDM (r = −0.45, −0.78, −0.39, and −0.58, respectively). The traits evaluated in the germination paper did not correlate with the traits evaluated in the greenhouse. Regarding the greenhouse experiments, the RDM was correlated in both experiments (r = 0.43). RDM_Exp_SS also showed a positive correlation with SPC_Exp_S, SDM_Exp_S, and SDM_Exp_SS (r = 0.41, 0.52, 0.41, respectively). In turn, RDM_Exp_S obtained a positive correlation with SPC_Exp_S, SPC_Exp_SS and SDM_Exp_SS (r = 0.41, 0.48, and 0.43, respectively).

Based on the PCA and hierarchical clustering UPGMA, a wide distribution of treatments for agronomic traits and P content was observed (Figure 1a,c). The inoculation of strains I12, I13, and I04 favored greater increments for most of the traits evaluated in maize in all experiments (germination paper, greenhouse_sand, and greenhouse_sand:soil). Strains I17, I05, I03, I16, I19, I21, I24, I01, I02, I09, and I26 led to better results for most traits in the greenhouse experiments. However, they showed the lowest values in the experiment on germination paper. Among these strains, I17 stood out, obtaining high SDM_Exp_SS, SPC_Exp_SS, and RDM_Exp_S values. Strains I20, I14, I23, I07, I22, I11, I18, I10, I06, I25, I08, and I15 provided greater increases in maize in the germination paper experiment. Based on these results, strains I13 and I17 were selected for further experiments and were called Ag87 and Ag94.

### 3.2. Genomic Analysis of Strains Ag87 and Ag94

The CLC Genomics Workbench 11 and IDBA Hybrid genome assembly strategies demonstrated the best results for assembly. First, a BLASTn search was performed using the largest contig to find a reference genome to be used in CONTIGuator. The strains CP001982 from *Bacillus megaterium* strain DSM319 and CP067341 from *Lysinibacillus agricola* strain FJAT-51161 were selected to align the contigs and generate the pseudoconting (scaffold) using CONTIGuator. The scaffolds contained 27 and 20 gaps, respectively, which were aligned against the raw sequencing reads using Bowtie2 and then evaluated and cured in CLC Genomics Workbench 11. In the genome of Ag87, two plasmids of 67,247 and 48,132 bp were found. The genomes of strains Ag87 and Ag94 showed read alignment rates of 93.44 and 90.53%, respectively, with sizes of 6,184,617 and 4,623,298 bp, respectively. Genome annotation via RAST verified a GC content of 38.1% for strain Ag87, with 6477 CDSs. From this total, 15 CDSs were related to rRNA and 119 to tRNA sequences (Figure S1). For strain Ag91, a GC content of 36.9% and 4709 CDSs were found, with 16 of those related to rRNA and 61 to tRNA sequences (Figure S1).

**Figure 2:**
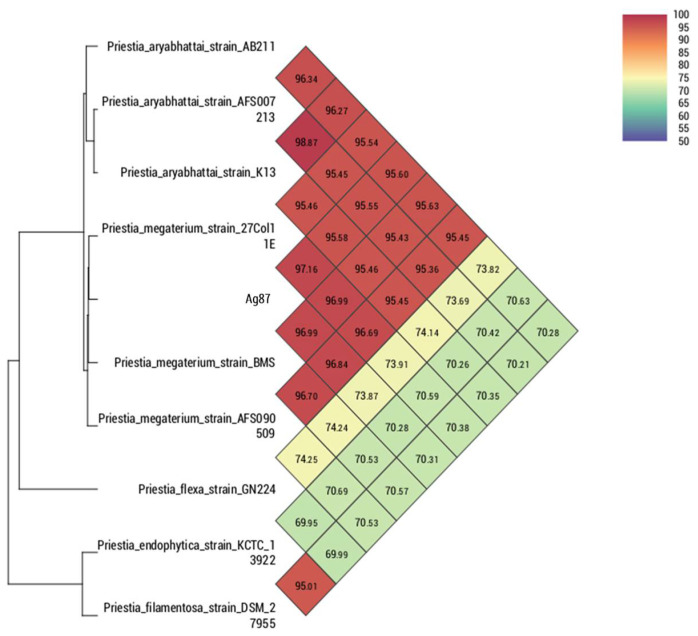
Heatmap generated with OAT software indicating the OrthoANI values of *Bacillus megaterium* strain Ag87 and the closely related *Bacillus* species.

**Figure 3:**
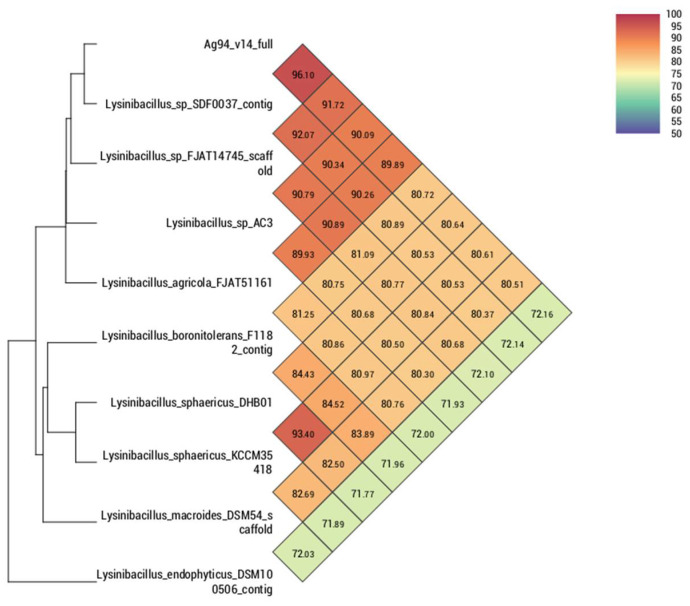
Heatmap generated with OAT software indicating the OrthoANI values of *Lysinibacillus* sp. strain Ag94 and closely related *Lysinibacillus* sp. species.

Comparing the strain Ag87 (GenBank accession CP098610) with the main species of the genus *Priestia*, it was observed that the average nucleotide identity (ANI) and digital DNA–DNA hybridization (dDDH) were higher with the groups of isolates of the *Priestia megaterium* species, ranging from 96.84 to 97.16% for ANI. For the strain Ag94 (GenBank accession CP096780), a greater genetic similarity was observed with the species of the genus *Lysinibacillus*. However, it was impossible to determine the species since the ANI values were <95% compared to the genomes of isolates with a defined species. The genome of the strain *Lysinibacillus* sp. SDF0037 obtained a value of 96.10% for ANI, suggesting that both belong to the same species. Nevertheless, deeper analyses are necessary to define a new species within the genus *Lysinibacillus*. In the comparison performed with orthoANI/GGDC, it was observed that strains Ag87 and Ag94 are located within the cluster containing most species of *Priestia megaterium* (Figure 2) and *Lysinibacillus* sp. (Figure 3), respectively. The circular genomes of the two strains are represented in Figures S2 and S3.

### 3.3. Field Data—Harvest 2020/2021

Based on the analysis of variance, a significant effect was observed for all sources of variation (environment, treatments, and environment × treatments) for grain yield (Table 1). The experimental coefficient of variation was 9.9, and all the assumptions of the analysis of variance were met (normality of errors, homogeneity of variance, and independence of errors). Among the environments, Londrina obtained the highest average yield, followed by Maringá, Entre Rios, and Guarapuava.

**Figure 4:**
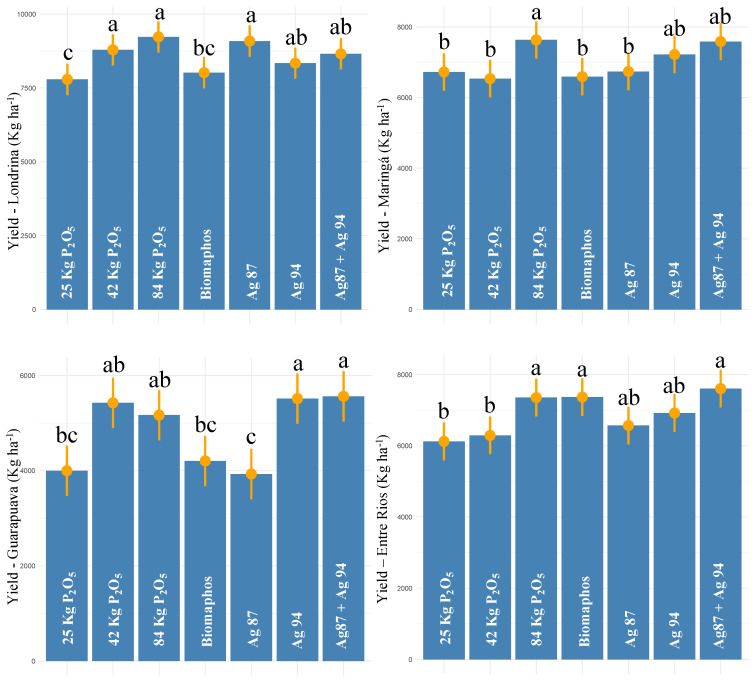
Tukey comparison (*p* < 0.05) for grain yield in maize experiments (first harvest—2020/2021) with seeds inoculated with different phosphorus-solubilizing bacteria at four sites in Paraná state—Brazil. (The same letter do not differ statistically at 5% probability by the Tukey test).

In Londrina, the highest yield was observed for 100% P (9228 kg ha^−1^), followed by the Ag87 (9088 kg ha^−1^), 50% P (8791 kg ha^−1^), Ag87 + Ag94 (8653 kg ha^−1^), and Ag94 (8339 kg ha^−1^) treatments (Figure 4). Compared with the 30% P control, the strains Ag87, Ag94, and Ag87 + Ag94 obtained yield increases of 16.6, 7.07, and 11.1%, respectively. For Maringá, the highest yields were observed for 100% P, Ag87 + Ag94, and Ag94, with 7632, 7587, and 7220 kg ha^−1^, respectively. These two biological treatments (Ag 94 and Ag87 + Ag94) showed yield increases of 7.3 and 12.8%, respectively, compared to the 30% P control.

In Guarapuava, higher yield values were also observed for the 50% P, 100% P, Ag94, and Ag87 + Ag94 treatments, with 5427, 5167, 5514, and 5561 kg ha^−1^, respectively. The strain Ag94 and the Ag87 + Ag94 combination obtained an average increase of 38% in productivity compared to the control (30% of P). For Entre Rios, the highest yields were observed for the Ag87 + Ag94, Biomaphos, 100% P, Ag94, and Ag87 treatments, with 7604, 7366, 6918, and 6566 kg ha^−1^, respectively. These treatments obtained 24, 20, 20, 13, and 7% productivity increases, respectively. Based on these experiments, strain Ag94 and its combination with Ag87 obtained average yield increases of 16.3 and 21.45%, respectively, at the four sites compared to the 30% P control. Furthermore, these strains did not differ statistically from the 100% P control.

For the phosphorus use efficiency indices (PUpE_g, PUtE_g, and PUE_g), significant effects were observed for all sources of variation (environments, treatments, and environments × treatments). The coefficients of variation were 12.8, 14.1, and 11.7, respectively. For PUpE_g, the Ag94 and Ag87 + Ag94 treatments obtained the highest values for Londrina and Maringá, while for Guarapuava, the highest values came from Biomaphos and Ag87 (Table 2). For Entre Rios, the highest values were observed for the Biomaphos, Ag87, Ag94, Ag87 + Ag94, and 30% P treatments.

Differences between treatments for PUtE_g were observed only in Londrina and Entre Rios. However, no differences were observed in the application of biological products compared to the 30% P control, indicating the non-influence of biological products in relation to the phosphorus utilization efficiency. For PUE_g, only in Guarapuava was a difference between biological products observed in relation to the 30% P control, with higher values obtained for Biomaphos, Ag87, and Ag87 + Ag94.

### 3.4. Field Data—Second Harvest 2021

**Figure 5:**
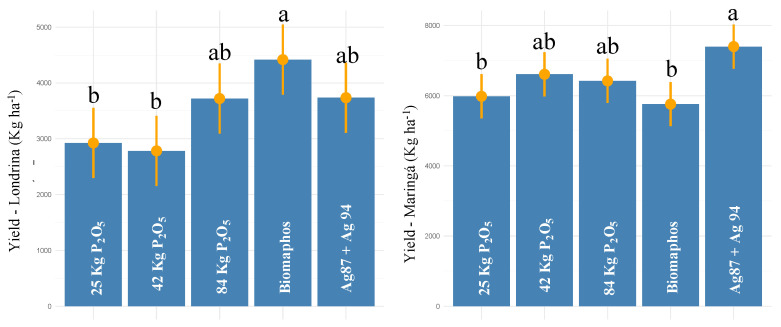
Tukey comparison (*p* < 0.05) for grain yield in maize experiments (second crop—2021) with seeds inoculated with different phosphorus-solubilizing bacteria at two sites in Paraná state—Brazil. (The same letter do not differ statistically at 5% probability by the Tukey test).

For the experiments installed in Paraná (Londrina and Maringá), a significant effect was observed for all sources of variation (treatments, environments, and treatments × environments) for grain yield (Table 2). The experimental coefficient of variation was 12.6, and all the assumptions of the analysis of variance were met. The average yield was 3516.6 kg ha^−1^ in Londrina and 6434.40 kg ha^−1^ in Maringá. Regarding the treatments, Biomaphos, 100% P, and Ag87 + Ag94 resulted in the highest yields (4419, 3722, and 3736 kg ha^−1^, respectively) in Londrina. By contrast, in Maringá, the highest yields were obtained by applying Ag87 + Ag94, 50% P, and 100% P (7398, 6611, and 6421 kg ha^−1^, respectively) (Figure 5).

**Figure 6:**
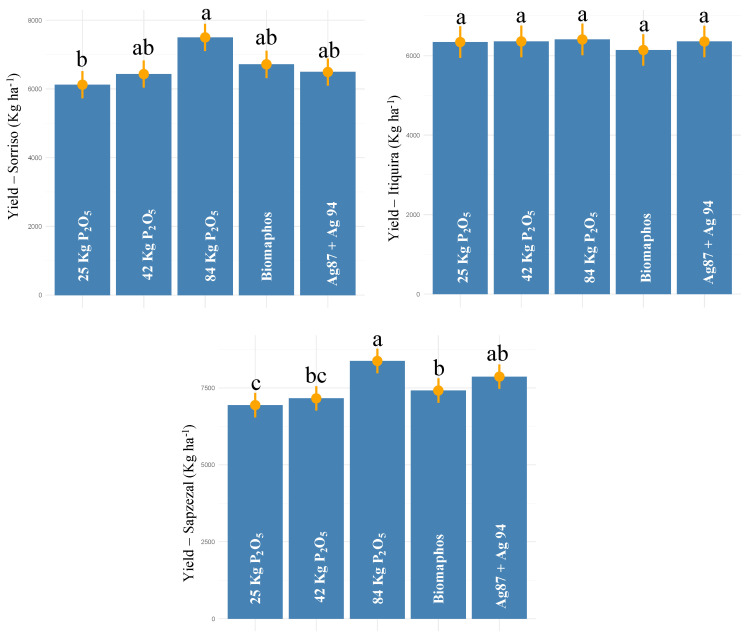
Tukey comparison (*p* < 0.05) for grain yield in maize experiments (second harvest—2021) with seeds inoculated with different phosphorus-solubilizing bacteria at three sites in Mato Grosso state—Brazil. (The same letter do not differ statistically at 5% probability by the Tukey test).

For the experiments conducted in Mato Grosso (Sorriso, Itiquira, and Sapezal), a significant effect was also observed for all sources of variation for grain yield (Table 2). The average productivity obtained by the environments was 6653.19, 6321.4, and 7553.43 kg ha^−1^, respectively (Figure 6). For Sorriso, the highest yield was obtained for 100% P (7500 kg ha^−1^), not differing statistically from Biomaphos, Ag87 + Ag94, and 50% P (6716.1, 6494.6, and 6432.2 kg ha^−1^, respectively). Biomaphos and Ag87 + Ag94 led to productivity increases of 9 and 6% in relation to the 30% P control, respectively. In Itiquira, no differences were observed between treatments, while for Sapezal, the highest productivity values were observed for the 100% P and Ag87 + Ag94 treatments (8377.5 and 7869.6 kg ha^−1^, respectively). The application of Ag87 + Ag94 resulted in an average yield increase of 13% in relation to the 30% P control.

## 4. Discussion

Phosphorus-solubilizing and plant growth-promoting rhizobacteria (PSB-PGPR) have gained prominence in world agriculture due to their beneficial effects on P use efficiency and improvement in radicular P acquisition [8,35]. To explore the biodiversity of microorganisms already adapted to the edaphoclimatic conditions in Brazil, we selected bacteria from the maize rhizosphere. Then, we performed various tests to choose bacterial strains with the potential for application as biological products capable of increasing P uptake efficiency and promoting plant growth.

For this purpose, rhizobacteria with the ability to solubilize phosphate and produce high amounts of IAA were selected. IAA plays an important role in root development, mainly in root hair and lateral root formation, improving nutrient absorption [36,37]. In this context, this combination, in addition to providing phosphate to plants, also stimulates the development of the organ, favoring greater capture of phosphorus. Kudoyarova et al. [36] verified that the *Paenibacillus illinoisensis* IB 1087 and *Pseudomonas extremeustralis* IB-Ki-13-1A strains, selected based on IAA production and phosphate solubilization, contributed efficiently to wheat root system development, favoring greater accumulation of biomass and phosphorus. Etesami et al. [38] found that the trait IAA plays an important role in selecting plant growth-promoting bacteria in rice. In the present study, 70% of the evaluated strains increased root development compared to the control in the germination paper experiment.

In the greenhouse experiments, the efficiency of bacterial strains on maize root development was lower than that in the tests conducted on germination paper. The inoculation efficiency of plant growth-promoting bacteria can vary according to the plant genotype, plant development stage, bacterial strain, and environmental conditions, which impact colonization and interaction with the plant [37]. This fact is corroborated when analyzing the greenhouse experiments, in which there was greater effectiveness of bacterial strains evaluated in sand:soil conditions. The lower efficiency of PSB-PGPR inoculation in the sand experiment may be related to the nutritional conditions the plant was subjected to, disfavoring an effective interaction with maize. On the other hand, in the sand:soil experiment, soil nutrients may have favored this interaction, reflecting a greater promotion of the root system and higher shoot P content. Furthermore, similar to plant–endophyte interactions, the “balanced antagonism” hypothesis can be applied to plant and PGPR interactions [39,40], where phenotypic plasticity in host plants can range from mutualism to antagonism, depending on the plant genotype, environmental conditions, and bacterial isolate.

Based on the studies carried out on germination paper and in a greenhouse (sand and sand:soil), four bacterial strains (I04, I12, I13, and I17) demonstrated a high potential for maize root growth and shoot P content, indicating promising PSB-PGPR. Based on this information, strains I13 (Ag87) and I17 (Ag94) were selected for genomic studies and evaluation of their potential under field conditions. The strain Ag87 was identified as *Bacillus megaterium*. This species is commonly found in soils and is a member of the microbiome of several host plants, acting mainly as PGPR [10,41,42]. In addition, strains of this species produce a wide range of bioactive compounds that promote plant growth and nutrient solubilization, mainly P and potassium [43,44]. Zhao et al. [45] found that the application of *B. megaterium* increased cucumber yield and improved soil phosphorus and potassium bioavailability.

The strain Ag94 was identified as *Lysinibacillus* sp. However, it was impossible to determine the species due to the ANI values (lower < 95%) compared to the genomes of isolates with a defined species. In this context, this strain may be a new species; therefore, further research is needed for this validation. *Lysinibacillus* species, previously described as members of the genus *Bacillus* [46], have 37 described species (http://www.bacterio.net/lysinibacillus.html, accessed on 11 April 2022). Most species of this genus are isolated from soil environments, and several works have demonstrated their potential as PSB-PGPR [47,48,49]. Evaluating several strains from the rice rhizosphere, Lelapalli et al. [49] found that *Lysinibacillus pakistanensis* PCPSMR15 has a high capacity for phosphate solubilization and growth promotion in beans, indicating that it is an important strain in the development of a commercial inoculant.

Under field conditions (2020/2021 harvest), the strains Ag87 and Ag94 obtained higher average yield increases than the 30% P control. In turn, when analyzed in combination, the average increase was even higher, indicating a positive effect when combining these strains. This fact is also corroborated for PUpE_g since the combined action of the strains increased P uptake efficiency compared to the control. In addition, the non-differentiation of inoculation (Ag87 + Ag94) with 100% P control indicates the possibility of reducing the P applied in maize. This reduction has great relevance for Brazilian agriculture due to the country’s high dependence on imported fertilizers and the increase in the costs of phosphate fertilizers in recent years [50].

The results of increased grain yield in maize when inoculated with the strains Ag87 + Ag94 were also verified in the experiments carried out in the second harvest under different weather conditions. Therefore, this is the first study that demonstrates the efficiency of the combined action of *Bacillus megaterium* and *Lysinibacillus* in maize, aiming at increasing yield and phosphorous uptake efficiency, indicating the potential of these strains in developing commercial inoculants for Brazilian agriculture.

## 5. Conclusions

The combined selection of strains capable of phosphate solubilization and indole-acetic acid production allowed obtaining bacteria with the potential to promote maize growth. The strains I13 (Ag87) and I17 (Ag94) selected as PSB-PGPR were identified as *Bacillus megaterium* and *Lysinibacillus* sp., respectively. The combined inoculation of these strains increased maize grain yield and phosphorous use efficiency, indicating the potential of these strains to be used as commercial inoculants for Brazilian agriculture. Further studies are needed to evaluate the effect of these strains on other crops of agricultural importance. In addition, the optimization of the bioprocess for industrial production of these strains is needed.

## Figures and Tables

**Table 1:** Analysis of variance for grain yield in maize experiments conducted in the first and second harvests (2020/2021 and 2021, respectively) with seeds inoculated with different phosphorus-solubilizing bacteria.

Source of Variation	First Harvest 2020/2021 Paraná		Second Harvest 2021 Paraná		Second Harvest 2021 Mato Grosso	
	DF	MS (Yield)	DF	MS (Yield)	DF	MS (Yield)
Repetitions/E	12	1,077,861	6	471,722	9	304,887
Environment (E)	3	65,585,288 **	1	85,089,692 **	2	8,320,357 **
Treatments (T)	6	3,104,344 **	4	1,443,243 *	4	2,022,543 **
E × T	18	837,210 *	4	1,970,383 **	8	563,600 **
Error	81	455,739	24	394,322	36	156,392
CV(%)		9.90		12.62		7.78
Mean Londrina		8558.09		3516.60		-
Mean Maringá		7004.05		6434.40		-
Mean Guarapuava		4828.13		-		-
Mean Entre Rios		6888.12		-		-
Mean Itiquira		-		-		6321.41
Mean Sorriso		-		-		6653.19
Mean Sapezal		-		-		7553.43
Shapiro–Wilk		0.98 ^ns^		0.97 ^ns^		0.98 ^ns^
Bartlett		31.10 ^ns^		22.60 ^ns^		14.55 ^ns^
Durbin–Watson		2.24 ^ns^		2.63 ^ns^		2.51 ^ns^

ns, **, and * indicate non-significance and significance at levels of 1 and 5% probability by the F test, respectively.

**Table 2:** Tukey comparison (*p* < 0.05) for phosphorus uptake efficiency (PUpE_g), phosphorus utilization efficiency (PUtE_g), and phosphorus use efficiency (PUE_g) in maize experiments (first harvest—2020/2021) with seeds inoculated with different phosphorus-solubilizing bacteria at four sites in Paraná state—Brazil.

Treatments	Londrina ^1/^			Treatments	Maringá		
	PUpE_g	PUtE_g	PUE_g		PUpE_g	PUtE_g	PUE_g
25 kg P_2_O_5_	1.31 b	395 ab	516 a	25 kg P_2_O_5_	0.72 b	757 a	545 ab
42 kg P_2_O_5_	0.78 c	494 a	385 b	42 kg P_2_O_5_	0.47 c	689 a	323 b
84 kg P_2_O_5_	0.45 d	382 ab	172 c	84 kg P_2_O_5_	0.23 d	698 a	160 c
Biomaphos	0.92 b	489 a	451 ab	Biomaphos	0.71 b	808 a	573 ab
Ag 87	1.27 b	475 a	603 a	Ag 87	0.76 b	552 a	420 b
Ag 94	1.38 a	412 ab	568 a	Ag 94	0.90 a	593 a	533 ab
Ag 87 + 94	1.58 a	347 b	548 a	Ag 87 + 94	0.80 ab	790 a	632 a
Treatments	Guarapuava			Treatments	Entre Rios		
	PUpE_g	PUtE_g	PUE_g		PUpE_g	PUtE_g	PUE_g
25 kg P_2_O_5_	0.42 c	1836 a	771 b	25 kg P_2_O_5_	0.50 ab	1908 a	954 a
42 kg P_2_O_5_	0.44 c	1726 a	759 b	42 kg P_2_O_5_	0.34 b	1328 b	451 c
84 kg P_2_O_5_	0.15 d	1242 a	186 c	84 kg P_2_O_5_	0.19 c	1604 a	304 c
Biomaphos	0.79 a	1334 a	1053 a	Biomaphos	0.73 a	1372 b	1001 a
Ag 87	0.78 a	1638 a	1278 a	Ag 87	0.59 ab	1513 b	892 b
Ag 94	0.35 c	2012 a	704 b	Ag 94	0.52 ab	1964 a	1021 a
Ag 87 + 94	0.61 b	1763 a	1075 a	Ag 87 + 94	0.63 ab	1985 a	1250 a

^1/^ Means followed by the same letter in the column do not differ statistically at 5% probability by the Tukey test.

## Data Availability

The datasets generated during and analyzed during the current study are available from L.S.A.G on reasonable request.

## Supplementary Materials

The following supporting information can be downloaded at: https://www.mdpi.com/article/10.3390/microorganisms10071401/s1: Table S1. Characterization of the four environments used in the 2020/2021 maize experiments. Table S2. Characterization of the five environments used in the 2021 maize experiments. Table S3. Scott–Knott mean clustering for the variables average root diameter (ARD), root surface area (RSA), root length (RL), root dry mass (RDM), and shoot dry mass (SDM) in maize seeds inoculated with phosphorus-solubilizing bacteria on germination paper. Table S4. Scott–Knott mean clustering for root dry mass (RDM), shoot dry mass (SDM) and shoot phosphorus content (SPC) in maize seeds inoculated with phosphorus-solubilizing bacteria in a greenhouse experiment with two different substrates (sand and sand:soil). Figure S1. SEED classification of Ag87 and Ag94 genomes. Pie chart depicting functional categories in the (a) Ag87 and (b) Ag Ag94 genomes. The SEED annotated genome was compared to hundreds of genomes maintained within the SEED integration. RAST annotation (server possessed identified protein encoding genes (PEGs), RNA genes, and repeat regions) was used to create the pie chart. Figure S2. Circular representation of the genome of *Bacillus megaterium* strain Ag87 using the program BRIG. From the inside to the outside, the legends are as follows: GC content, GC skew and representation of the genomes of *Bacillus megerium* strains: 27Col11E, AB211, BMS, RIT381, KLBMP_4941, GN 224, PKS_39, Hbc603, KCTC_13922, and 36172C. Figure S3. Circular representation of the genome of *Lysinibacillus* sp. strain Ag94 using the program BRIG. From the inside to the outside, the legends are as follows: GC content, GC skew and representation of the genomes of *Lysinibacillus* sp. strains: SDF0037, FJA14745, JNUCC51, KCTC33748, AC3, F1182, N80, HST98, NGB1202, S655, TC37, KCGM35418, DSM54, GV32, MF12, WS4626, DSN100506, and Marseille P5727.
