# Supplementary material for: Efficiency of Combining Strains Ag87 (Bacillus megaterium) and Ag94 (Lysinibacillus sp.) as Phosphate Solubilizers and Growth Promoters in Maize
Source: Microorganisms. 2022 Jul 12;10(7):1401. doi: 10.3390/microorganisms10071401 (PMC9315647; doi:10.3390/microorganisms10071401)
Supplement: Supplementary file 1 [file microorganisms-10-01401-s001.zip › microorganisms-1774043-supplementary.pdf]

microorganisms

MDPI

Article

Efficiency of combining strains Ag87 (*Bacillus megaterium*) and Ag94 (*Lysinibacillus* sp.) as phosphate solubilizers and growth promoters in maize

Luana Rainieri Massucato[1]. Suelen Regina de Araújo Almeida[1]. Mayara Barbosa Silva[2]. Mirela Mosela[3]. Douglas Mariani Zeffa[4]. Alison Fernando Nogueira[1]. Renato Barros de Lima Filho[4]. Silas Mian[1]. Allan Yukio Higashi[1]. Gustavo Manoel Teixeira[3]. Gabriel Danilo Shimizu[1]. Renata Mussoi Giacomin[5]. Ricardo Cancio Fendrich[2]. Marcos Ventura Faria[6]. Carlos Alberto Scapim[4] and Leandro Simões Azeredo Gonçalves[1.4*]

[1]Agronomy Department. Universidade Estadual de Londrina (UEL). Londrina. Paraná. Brazil.
[2]NODUSOJA. Colombo. Paraná. Brazil.
[3]Microbiology Department. Universidade Estadual de Londrina (UEL). Londrina. Paraná. Brazil.
[4]Agronomy Department. Universidade Estadual de Maringá (UEM). Maringá. Paraná. Brazil.
[5]Biology Department. Universidade Estadual do Centro Oeste (Unicentro). Guarapuava. Paraná. Brazil
[6]Agronomy Department. Universidade Estadual do Centro Oeste (Unicentro). Guarapuava. Paraná. Brazil.
*Correspondence: leandrosag@uel.br

Table S1. Characterization of the four environments used in the 2020/2021 maize experiments.

Characteristics[1/]	Londrina	Maringá	Guarapuava	Entre Rios do Oeste
Geographical coordinates	23° 17′ S; 51° 10′ W	23° 11′ S; 52° 03′ W	25° 23′ S; 51° 29′ W	24° 68′ S; 54 ° 28′ W
Altitude (m)	550	555	1026	230
Climate[2/]	Cfa	Cfa	Cfb	Cfa
Soil	Dystroferric Red Latosol	Dystroferric Red Latosol	Dystroferric Bruno Latosol	Eutroferric Red latosol
pH ($CaCl_2$)	5.2	5.2	4.5	4.7
H+Al (cmolc dm^3)	3.3	3.2	5.5	4.3
K (cmolc dm^3)	0.8	0.5	0.3	0.6
Ca (cmolc dm^3)	5.4	2.9	2.1	4.8
Mg (cmolc dm^3)	1.6	1.3	0.6	1.7
Al (cmolc dm^3)	0.1	0.0	0.9	0.2
P (mg dm^3)	22.6	10.7	7.0	20.0
Organic Matter (%)	2.8	1.8	4.2	2.9

[1/] Physical-chemical analyses were performed using soil layer samples from 0 to 20 cm.

[2/] Köppen climate classification = Cfa. Humid subtropical climate; Cfb: Temperate oceanic climate.

Table S2. Characterization of the five environments used in the 2021 maize experiments.

Characteristics[1/]	Londrina - Paraná	Maringá - Paraná	Itiquira – Mato Grosso	Sorriso – Mato Grosso	Sapezal – Mato Grosso
Geographical coordinates	23° 17′ S; 51° 10′ W	23° 11′ S; 52° 03′ W	17°29′35″ S 54°45′29° W	12°29′26″ S 55°35′15°W	13°29′11″ S; 58°54′ 32° W
Altitude (m)	550	555	490	385	570
Climate[2/]	Cfa	Cfa	Cfa	Cfa	Cfa
Soil	Dystroferric Red Latosol	Dystroferric Red Latosol	Latosol with a clayey texture	Latosol with a clayey texture	Latosol with a clayey texture
pH ($CaCl_2$)	5.4	5.7	5.8	4.5	5.2
H+Al (cmolc dm^3)	3.2	3.1	5	5.6	4
K (cmolc dm^3)	0.9	0.7	0.28	0.15	0.01
Ca (cmolc dm^3)	4.8	3.2	2.7	1.2	2.8
Mg (cmolc dm^3)	2.1	2.3	1.1	0.5	1.1
Al (cmolc dm^3)	0.1	0.0	0.0	0.2	0.0
P (mg dm^3)	11.6	9.2	9.5	2.4	5.6
Organic Matter (%)	3.1	2.0	3.5	3.2	2.5

[1/] Physical-chemical analyses were performed using soil layer samples from 0 to 20 cm.

[2/] Köppen climate classification = Cfa. Humid subtropical climate; Cfb: Temperate oceanic climate.

Table S3. Scott–Knott mean clustering for the variables average root diameter (ARD). root surface area (RSA). root length (RL). root dry mass (RDM). and shoot dry mass (SDM) in maize seeds inoculated with phosphorus-solubilizing bacteria on germination paper.

Treatments	Traits Evaluated				
	ARD[1/]	RSA	RL	RDM	SDM
Control	0.0707 b	119.31 d	723.65 c	0.1749 d	0.2412 b
Biomaphos	0.0743 b	113.85 d	678.27 c	0.1661 d	0.2294 c
Nodugram	0.0831 a	134.05 d	797.18 b	0.1877 c	0.2481 b
Strain01	0.0691 b	111.94 d	720.37 c	0.1728 d	0.2277 c
Strain02	0.0695 b	114.45 d	682.55 c	0.1808 d	0.2455 b
Strain03	0.0744 b	113.32 d	691.49 c	0.1805 d	0.2368 b
Strain04	0.1054 a	200.40 a	973.19 a	0.2482 b	0.2258 c
Strain05	0.0770 b	125.09 d	769.89 b	0.2024 c	0.2210 c
Strain06	0.0999 a	174.55 b	793.92 b	0.2262 b	0.2183 c
Strain07	0.0855 a	137.68 d	609.99 d	0.2036 c	0.2425 b
Strain08	0.0912 a	166.62 c	744.56 c	0.2171 c	0.2025 d
Strain09	0.0577 c	91.26 e	558.32 d	0.1459 d	0.2778 a
Strain10	0.0914 a	192.17 a	880.50 a	0.2910 a	0.1629 d
Strain11	0.0769 b	144.41 c	921.80 a	0.2274 b	0.2242 c
Strain12	0.0944 a	169.15 c	814.32 b	0.2336 b	0.2238 c
Strain13	0.0889 a	181.86 b	812.01 b	0.2893 a	0.1846 d
Strain14	0.0934 a	152.49 c	688.05 c	0.2122 c	0.2488 b
Strain15	0.0893 a	164.49 c	711.90 c	0.2423 b	0.2002 d
Strain16	0.0836 a	120.79 d	753.80 b	0.1940 c	0.2450 b
Strain17	0.0592 c	85.92 e	572.14 d	0.1704 d	0.2398 b
Strain18	0.0894 a	173.16 b	821.10 b	0.2356 b	0.2325 b

Strain19	0.0817 a	133.17 d	804.67 b	0.2055 c	0.2180 c
Strain20	0.0812 a	143.22 c	867.83 a	0.2134 c	0.2907 a
Strain21	0.0568 c	96.41 e	619.58 d	0.1647 d	0.2565 b
Strain22	0.0932 a	157.45 c	739.88 c	0.2023 c	0.2474 b
Strain23	0.0947 a	151.52 c	682.99 c	0.2040 c	0.2584 b
Strain24	0.0693 b	107.54 d	666.50 c	0.1610 d	0.2487 b
Strain25	0.0849 a	159.92 c	688.31 c	0.2927 a	0.1818 d
Strain26	0.0769 b	118.29 d	528.76 d	0.1585 d	0.2938 a

[1/] Means followed by the same letter in the column do not differ statistically at 5% probability by the tukey's test.

Table S4. Scott–Knott mean clustering for root dry mass (RDM). shoot dry mass (SDM) and shoot phosphorus content (SPC) in maize seeds inoculated with phosphorus-solubilizing bacteria in a greenhouse experiment with two different substrates (sand and sand:soil).

Tratamentos	Experiment – Sand			Experiment – Sand:Soil (3:1)		
	RDM[1/]	SDM	SPC	RDM	SDM	SPC
Control	0.4976 b	0.5468 b	0.3214 b	0.8179 b	0.8718 a	0.4218 c
Pinsol	0.5316 b	0.7362 a	0.6640 a	0.7672 b	0.8814 a	0.6320 b
Biomaphos	0.6322 b	0.7121 a	0.6081 a	0.8750 b	0.9789 a	0.7980 a
Strain01	0.5942 b	0.7079 a	0.6101 a	0.8139 b	0.9761 a	0.6591 b
Strain02	0.6399 b	0.7108 a	0.6018 a	0.8514 b	0.9201 a	0.6241 b
Strain03	0.6440 b	0.7533 a	0.6015 a	1.0270 a	0.9908 a	0.6700 b
Strain04	0.8423 a	0.7283 a	0.6291 a	1.0216 a	1.1014 a	0.7299 a
Strain05	0.8034 a	0.7375 a	0.6112 a	0.9600 a	0.9077 a	0.6230 b
Strain06	0.6677 b	0.6626 b	0.6212 a	0.6653 b	0.7503 a	0.6442 b
Strain07	0.6097 b	0.7382 a	0.6382 a	0.9123 b	0.9916 a	0.6392 b
Strain08	0.6932 b	0.6726 b	0.5726 a	0.8797 b	0.9492 a	0.6920 b
Strain09	0.6394 b	0.7522 a	0.6615 a	0.8373 b	1.0216 a	0.6220 b
Strain10	0.6061 b	0.6458 b	0.5514 a	0.7473 b	0.8641 a	0.7641 a
Strain11	0.6131 b	0.6580 b	0.6110 a	0.8696 b	0.9688 a	0.6522 b
Strain12	0.6770 b	0.7586 a	0.6620 a	1.2650 a	0.9165 a	0.6571 b
Strain13	0.8930 a	0.7102 a	0.7051 a	1.0027 a	0.9647 a	0.7312 a
Strain14	0.5983 b	0.6042 b	0.6141 a	0.9646 a	0.9778 a	0.6512 b
Strain15	0.5821 b	0.5928 b	0.5920 a	0.7331 b	0.9705 a	0.6270 b
Strain16	0.7355 b	0.7746 a	0.6220 a	0.9065 b	0.9022 a	0.6999 b
Strain17	0.8167 a	0.7285 a	0.6401 a	1.0164 a	1.1175 a	0.7922 a
Strain18	0.5831 b	0.6739 b	0.6210 a	0.9384 a	1.0386 a	0.6345 b
Strain19	0.6475 b	0.7659 a	0.6514 a	0.9009 b	0.9628 a	0.6914 b

Strain20	0.6210 b	0.6210 b	0.6410 a	0.8220 b	0.9220 a	0.6620 b
Strain21	0.5827 b	0.6434 b	0.6011 a	0.8710 b	0.9372 a	0.6731 b
Strain22	0.6411 b	0.7017 a	0.6210 a	0.8530 b	0.9771 a	0.5961 b
Strain23	0.6180 b	0.6575 b	0.6610 a	0.8449 b	0.9555 a	0.7071 a
Strain24	0.6610 b	0.7010 a	0.6220 a	0.8014 b	0.9014 a	0.6921 b
Strain25	0.6103 b	0.7103 b	0.6234 a	0.8247 b	0.9848 a	0.61520 b
Strain26	0.6642 b	0.7642 b	0.6320 a	1.0324 a	0.9349 a	0.6012 b

[1/] Means followed by the same letter in the column do not differ statistically at 5% probability by the tukey`s test.

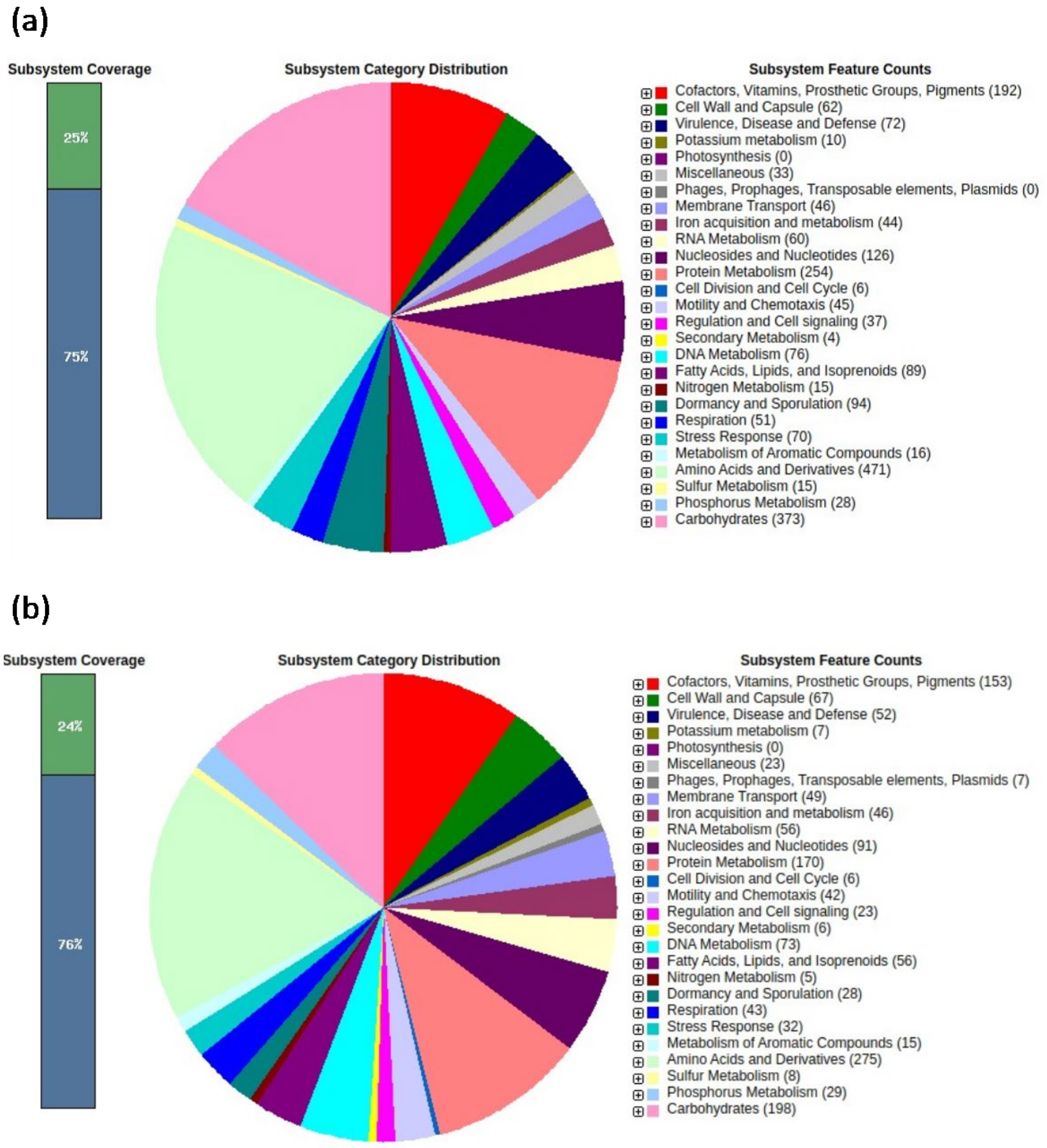

Figure S1. SEED classification of Ag87 and Ag94 genomes. Pie chart depicting functional categories in the (a) Ag87 and (b) Ag Ag94 genomes. The SEED annotated genome was compared to hundreds of genomes maintained within the SEED integration. RAST annotation (server possessed identified protein encoding genes (PEGs). RNA genes. and repeat regions) was used to create the pie chart.

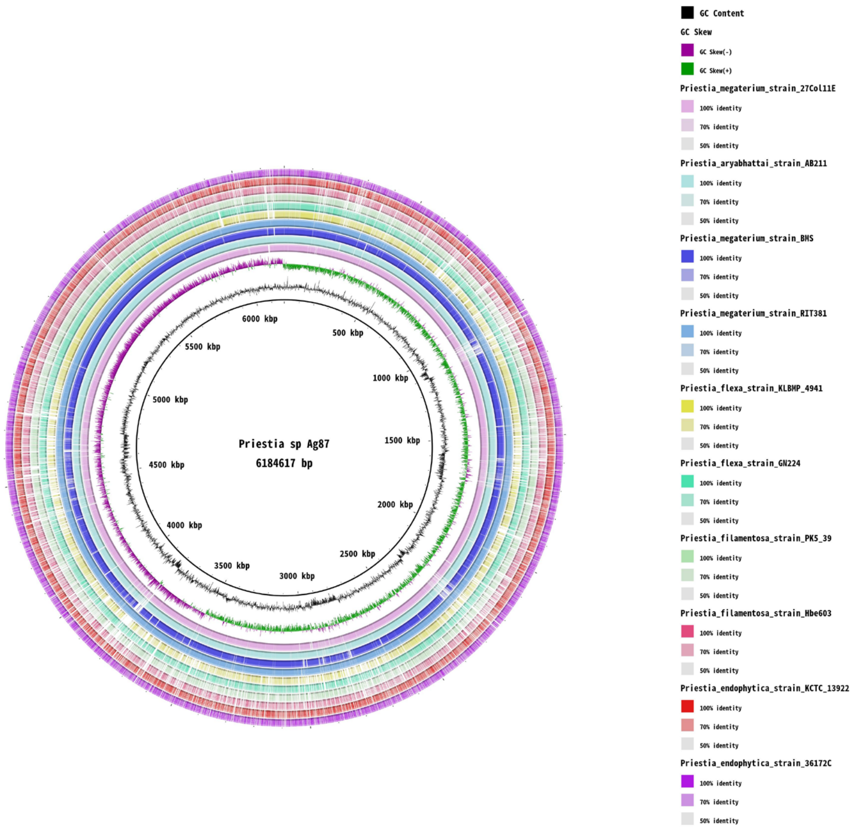

Figure S2. Circular representation of the genome of *Bacillus megaterium* strain Ag87 using the program BRIG. From the inside to the outside. the legends are as follows: GC content. GC skew and representation of the genomes of *Bacillus megerium* strains: 27Col11E. AB211. BMS. RIT381. KLBMP_4941. GN 224. PKS_39. Hbc603. KCTC_13922. and 36172C.

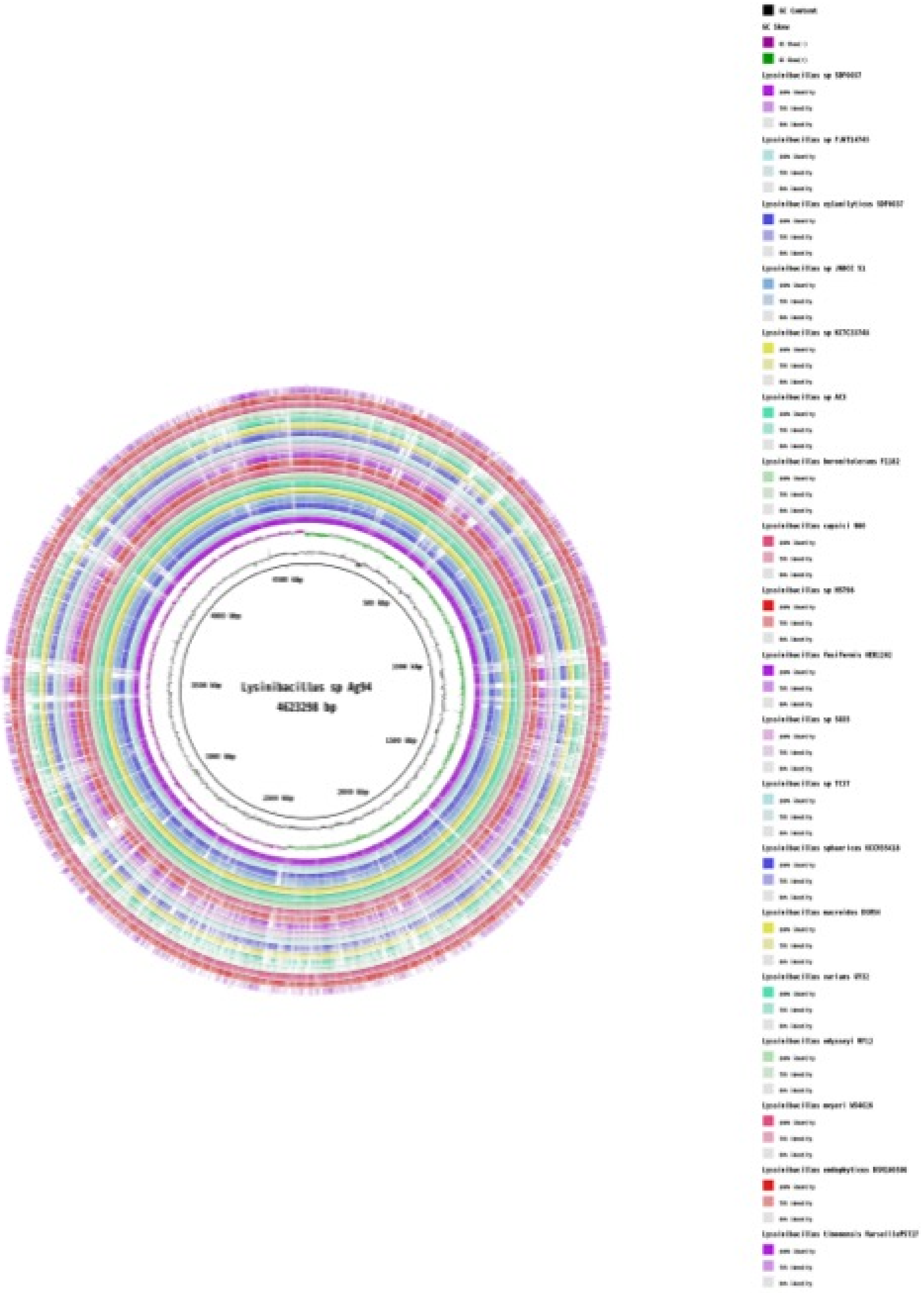

Figure S3. Circular representation of the genome of *Lysinibacillus* sp. strain Ag94 using the program BRIG. From the inside to the outside. the legends are as follows: GC content. GC skew and representation of the genomes of *Lysinibacillus* sp. strains: SDF0037. FJA14745. JNUCC51. KCTC33748. AC3. F1182. N80. HST98. NGB1202. S655. TC37. KCGM35418. DSM54. GV32. MF12. WS4626. DSN100506. and Marseille P5727.
